# Dynamical systems as a level of cognitive analysis of multi-agent learning: Algorithmic foundations of temporal-difference learning dynamics

**DOI:** 10.1007/s00521-021-06117-0

**Published:** 2021-06-23

**Authors:** Wolfram Barfuss

**Affiliations:** 1 School of Mathematics, University of Leeds, Leeds, UK; 2 Tübingen AI Center, University of Tübingen, Tübingen, Germany

**Keywords:** Multi-agent learning, Temporal-difference reinforcement learning, Evolutionary game theory, Levels of analysis

## Abstract

A dynamical systems perspective on multi-agent learning, based on the link between evolutionary game theory and reinforcement learning, provides an improved, qualitative understanding of the emerging collective learning dynamics. However, confusion exists with respect to how this dynamical systems account of multi-agent learning should be interpreted. In this article, I propose to embed the dynamical systems description of multi-agent learning into different abstraction levels of cognitive analysis. The purpose of this work is to make the connections between these levels explicit in order to gain improved insight into multi-agent learning. I demonstrate the usefulness of this framework with the general and widespread class of temporal-difference reinforcement learning. I find that its deterministic dynamical systems description follows a minimum free-energy principle and unifies a boundedly rational account of game theory with decision-making under uncertainty. I then propose an on-line sample-batch temporal-difference algorithm which is characterized by the combination of applying a memory-batch and separated state-action value estimation. I find that this algorithm serves as a micro-foundation of the deterministic learning equations by showing that its learning trajectories approach the ones of the deterministic learning equations under large batch sizes. Ultimately, this framework of embedding a dynamical systems description into different abstraction levels gives guidance on how to unleash the full potential of the dynamical systems approach to multi-agent learning.

## Introduction

*Multi-agent learning.* In a multi-agent system, a collective of autonomous agents interacts in a shared environment. Multi-agent systems are important for a variety of application domains, such as traffic [3, 90, 113], manufacturing [111], electricity [82] and finance [61]. Furthermore, a sound understanding of multi-agent systems is relevant for other fields, such as biology [68], economics [28], sustainability [10] and social sciences in general [19]. For artificial agents, preprogrammed agent behavior cannot lead to satisfactory solutions when the behavior of other agents and the shared environment is unknown or too complex. The agents need to learn an appropriate course of actions by themselves. Likewise, natural agents do use many forms of learning, which is required when the future is unpredictable and complex and therefore precise planning is doomed to failure.

While many learning mechanisms have been studied and applied in a multi-agent setting [31, 92], a significant amount of work concerns multi-agent reinforcement learning [17, 105]. Reinforcement learning is a trial-and-error method of mapping situations to actions in order to maximize a numerical reward signal [98]. Particular challenging is the case when rewards come as a delayed consequence of current actions. For this case, the class of reinforcement learning procedures that utilizes the difference between estimates made from past and current experiences, called *temporal-difference learning* [96], has been particularly influential. Also, remarkable similarities exist between computational reinforcement learning and results of neuroscientific experiments [24]. Dopamine appears to convey temporal-difference errors to brain structures where learning and decision-making take place [87]. It has further been argued that this dopamine reward-prediction error signal constitutes a potential neuronal substrate for the crucial economic decision variable of utility [88].

Reinforcement learning algorithms can be divided into two categories: model-free and model-based. *Model-free* methods learn directly from experience, whereas *model-based* methods learn a model of the environment to plan their course of actions [44]. In multi-agent settings, agents can be further categorized into two categories [15, 105]: *independent learners*, which learn the value of situation-action mappings only for their own actions and *joint-action learners*, where the learning takes place over the joint-action space [20].

*Challenges.* However, the application of multi-agent learning systems is hindered by numerous challenges:

*Complexity.* The joint state-action space suffers from the curse of dimensionality, hindering the scalability of algorithms to realistic problem sizes [17]. While deep-learning techniques can enable the scalability of multi-agent learning, several new challenges need to be addressed regarding implementation, computational demands and scientific reproducibility [43].

*Non-stationarities.* Each agent’s environment becomes non-stationary due to the other learning agents. Thus, each agent is faced with a moving-target problem [105] which invalidates the convergence properties of most single-agent learning algorithms [17, 42]. Also, the exploration–exploitation trade-off is further complicated due to the presence of multiple agents. Agents need to explore to obtain information not only about the environment, but also about the other agents [17]. As a result, the analysis of the transient learning behavior gains in importance.

*Coordination needs.* Agents need to coordinate between equally good policies, since the effect of any agent’s action depends also on the actions taken by the other agents [17, 72]. Even more challenging is the case when the agents did not train together and have to coordinate in an ad hoc [95] or zero-shot [51] manner.

*Social dilemmas.* Social dilemmas can arise, typically defined as a situation in which any agent prefers the socially defecting choice, regardless of what the other agents do, yet all agents are better off if all choose the socially cooperative option [23].

*Learning goal.* Last, defining an appropriate learning goal still remains challenging since the objectives of the agents are not necessarily aligned [17, 93, 117].

Taken together, these challenges culminate in the challenge of the *interpretability* of multi-agent learning systems. Despite the lack of consensus on how to specify interpretable machine learning, it has been widely recognized as an important aspect for the advancement of autonomous learning systems [65]. The majority of interpretability research focuses on methods for classifying high-dimensional data. Techniques for interpretable reinforcement learning have not been extensively studied [45]. As Doshi-Velez and Kim [27] argue, the need for interpretability stems from an incompleteness in the problem formalization. As the list of challenges above highlights, multi-agent learning with various types of learners in various types of environments can find themselves in various types of strategic interactions and dynamic regimes under various types of learning goals. This openness of the multi-agent learning problem formulation highlights the need for interpretable multi-agent learning methods.

*Replicator Reinforcement Learning Dynamics.* A dynamical systems approach, based on the link between evolutionary game theory and reinforcement learning, contributes to solving these challenges by providing improved, qualitative insights into the emerging collective learning dynamics [15]. This link turned out to be beneficial for applications regarding hyper-parameter tuning [56, 58], the design of new reinforcement learning algorithms [36, 40], the creation of novel evaluation metrics [78] and the analysis of strategic interactions [116]. Therefore, it is a prime candidate for interpretable multi-agent learning research.

In their seminal work, Börgers and Sarin showed how one of the most basic reinforcement learning update schemes, cross-learning [22], can converge to the deterministic replicator dynamics of evolutionary games theory [18]. This connection opened up all the tools of dynamical systems theory to the study of collective learning. The relationship between the two fields is as follows: one population with a frequency over phenotypes in the evolutionary setting corresponds to one agent with a frequency over actions in the learning setting [103]. The convergence to the replicator dynamics has also been shown for stateless Q-learning [86, 104], following some mathematical limiting procedure.

Yet, there is ambiguity as to how this deterministic— sometimes also called *evolutionary *—limit is taken. Consequently, it remains also unclear how to interpret these dynamics of multi-agent reinforcement learning [6].

One technique sends the learning rate to zero, resulting in replicator-type dynamics in continuous time [86, 104]. However, previous work found that discrepancies arise between the learning trajectories of the limiting learning equations and the actual Q-learning algorithm at small learning rates. This policy-bias problem has been addressed by modifications of the basic Q-learning algorithm. The value of an action is updated proportional to the inverse of the frequency of choosing that action. This suggests that replicator dynamics learning actually matches a frequency-adjusted Q-learning algorithm [1, 55].

Alternatively, a deterministic limit of reinforcement learning can be taken in discrete time, resulting in a set of difference equations for the action probabilities [14, 32–34]. This technique assumes that agents update their action values and policies only once every *K* rounds, using averages of the collected rewards. Similar methods in computer science are known as experience replay [64] or batch learning [60]. Deterministic dynamics emerge by sending the size of the batch *K* to infinity.

While the construction of such a batch-learning algorithm is straight forward for stateless repeated games, it is less clear how learning dynamics and batch algorithm relate in multi-state environments. Overall, there is much less work on learning dynamics in changing environments. Multiple variants of state-coupled replicator dynamics [40, 41, 109] have been introduced, yet, all of these dynamics consider an average reward setting, whereas in temporal-difference learning a discounted reward is commonly used. Recently, we introduced an analytical method to derive the deterministic, discrete-time limit of temporal-difference reinforcement learning with discounted rewards [7]. This method extends on the idea of batch learning with an infinite batch size, yet, it is still an open question how a temporal-difference batch learning algorithm must be constructed such that the learning trajectories of the algorithm can approximate those of the deterministic dynamics under large batch sizes.

*Cognitive levels of analysis.* In order to resolve ambiguity around the interpretation of multi-agent learning using dynamical systems theory, I propose to embed the dynamical systems approach within multiple levels of cognitive analysis. Marr’s level of analysis—computational, algorithmic and implementation—has had far-reaching influence in both neuroscience and cognitive science [70, 71]. They correspond to an abstract characterization of the computational problem being solved, the algorithm executing that solution, and the hardware implementing that algorithm. This framework highlights that it is valid, fruitful, and even necessary to analyze cognition by forming abstraction barriers which result in different levels of analysis [35]. Griffiths and colleagues [35] proposed to define levels of analysis that lie between the computational and the algorithmic, leading to the study of the optimal use of cognitive resources [37, 50, 52, 63, 83].

*Contributions.* I take on this idea of different levels of analysis and the value of building bridges between them and explicitly apply it to multi-agent learning.

First, I associate the dynamical systems level—manifested by replicator-alike equations—to an algorithmic level of high abstraction. Note that dynamical systems approaches to agent–environment interactions in particular and cognitive science in general have a long tradition [12, 13]. In this article, I explore the connections between this dynamical systems level, the computational level and an algorithmic level of lower abstraction. Like Griffiths and colleagues [35], I do not deal with the implementation level.

Second, I establish connections between the replicator-alike reinforcement learning equations [7] and the computational level. For multi-agent learning, the computational level is characterized by the framework of stochastic games [66] which I introduce in Sect. 2. I conjecture the relationship between the learning dynamics and Markov Perfect equilibria, a solution concept of stochastic games, and show that the dynamics follow a minimum free-energy principle (Sect. 3).

Third, I connect the dynamical systems level to an algorithmic level of lower abstraction. To do so, I propose a novel temporal-difference batch-learning algorithm (Sect. 4) and show that the learning trajectories of the algorithm converge to the ones of the deterministic equations under increasing batch sizes (Sect. 5).

**Fig. 1:**
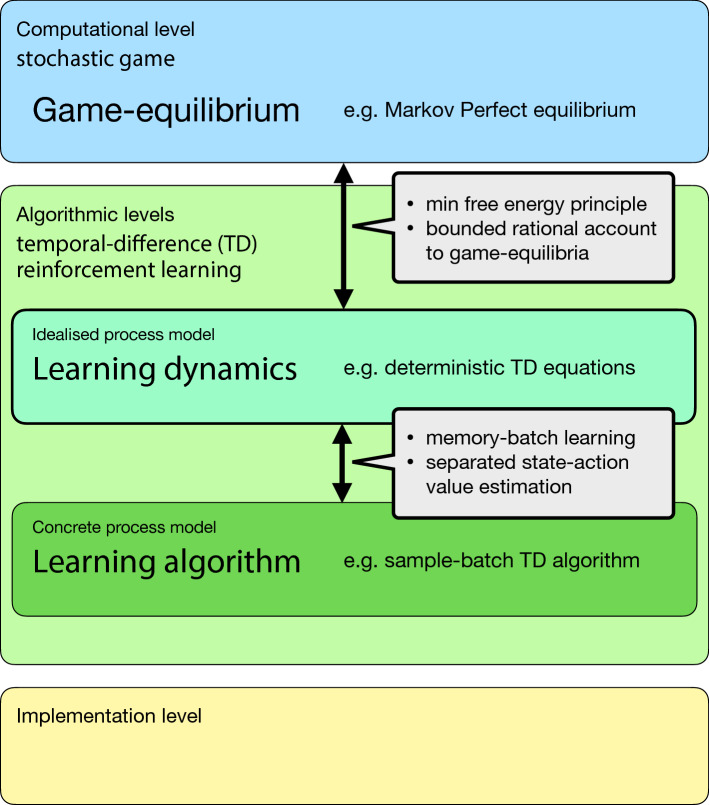
Levels of analysis of multi-agent learning. The dynamical systems level of analysis is embedded between the computational level and algorithmic levels of lower abstraction. The computational level is defined by the framework of stochastic games and related game-equilibrium concepts. Applying this concept, I find that the deterministic learning equations follow a minimum free energy principle and represent a bounded rational account of the game-equilibria. The proposed sample-batch temporal-difference algorithm is characterized by the combination of applying a memory-batch and separated state-action value estimation. Its learning trajectories converge to the ones of the deterministic learning equations under large batch sizes

I finish with a discussion about specifics, limitations and related work about the dynamic equations and the proposed sample-batch algorithm and highlight how the learning dynamics form a model of bounded rationality. I also give directions for future work and potential applications (Sect. 6). Fig. 1 shows a graphical overview of the paper’s main contributions.

## Background

### Stochastic games

Stochastic games are a formal model for multi-agent environment systems. They generalize both repeated normal form games and Markov decision processes (MDPs). MDPs are generalized by introducing multiple agents. Repeated games are generalized by introducing an environment with multiple states and transition probabilities between those states. All agents choose their actions simultaneously. The transition probabilities depend on the joint action and the current environmental state. So do the rewards. Formally, the game $G=\langle N, {\mathcal {S}}, \underline{{\mathcal {A}}}, T, {\underline{R}}, {\underline{X}} \rangle$ is a stochastic game with $N \in {\mathbb {N}}$ agents. The environment consists of $Z \in {\mathbb {N}}$ states ${\mathcal {S}}=(S_1, \dots , S_Z)$. In each state *s*, each agent *i* has $M \in {\mathbb {N}}$ available actions ${\mathcal {A}}^i = (A^i_1,\dots ,A^i_M)$ to choose from. $\underline{{\mathcal {A}}} = \prod _i {\mathcal {A}}^i$ is the joint-action set and agents choose their actions simultaneously.

The transition function $T: {\mathcal {S}} \times \underline{{\mathcal {A}}} \times {\mathcal {S}} \rightarrow [0, 1]$ determines the probabilistic state change. $T(s, {\underline{a}}, s')$ is the transition probability from current state *s* to next state $s'$ under joint action ${\underline{a}} = (a^1,\dots ,a^N) \in \underline{{\mathcal {A}}}$.

The reward function ${\underline{R}}: {\mathcal {S}} \times \underline{{\mathcal {A}}} \times {\mathcal {S}} \rightarrow {\mathbb {R}}^N$ maps the triple of current state *s*, joint action ${\underline{a}}$ and next state $s'$ to an immediate reward value for each agent. $R^i(s,{\underline{a}},s')$ is the reward agent *i* receives.

Agents choose their actions probabilistically according to their policy $X^i : {\mathcal {S}} \times {\mathcal {A}}^i \rightarrow [0,1]$. $X^i(s, a)$ is the probability that agent *i* chooses action *a* given the environment is in state *s*. ${\underline{X}}(s, {\underline{a}}) = \prod _i X^i(s, a^i)$ is the joint policy.

I chose an identical number of actions for all states and all agents out of notational convenience. With $${\underline{a}}^{-i} = (a^1,\dots ,$$ $$a^{i-1},$$ $$a^{i+1},$$ $$\dots ,$$ $$a^N)$$ I denote the joint action except agent *i*’s. Throughout this paper I only consider ergodic environments without absorbing states.

### Temporal-difference reinforcement learning

Temporal-difference Q-learning is one of the most widely used reinforcement learning algorithms [98, 112]. Agents successively improve their evaluations of the quality of the available actions at each state. At time step *t*, agent *i* evaluates action *a* in state *s* to be of quality $Q^i_t(s, a)$. Those state-action values $Q^i_t(s, a)$ are then updated after selecting action $a_t$ in state $s_t$ according to

1 $$\begin{aligned} Q^i_{t+1}(s_t,a_t) = Q^i_{t}(s_t,a_t) + \alpha \cdot \delta ^i_t(s_t,a_t), \end{aligned}$$

with the temporal-difference error

$$\begin{aligned} \delta ^i_t(s_t,a_t):= & {} (1-\gamma )r^i_t \\&+ \gamma \max _b Q^i_t(s_{t+1}, b) - Q^i_t(s_t,a_t). \end{aligned}$$

This update can be derived from the assumption that agent *i* aims to maximizes its expected discounted future reward $G^i_t = (1-\gamma )\sum _k^\infty \gamma ^k r^i_{t+k}$, where the *discount factor* parameter $\gamma \in [0,1)$ regulates how much the agent cares for future rewards. The pre-factor $(1-\gamma )$ normalizes the state-action values to be on the same numerical scale as the rewards. The *learning rate* parameter $\alpha \in (0,1)$ regulates how much new information is used for a state-action-value update. For the sake of simplicity, I assume identical parameters across agents throughout this paper and therefore do not equip parameters with agent indices. The variable $r^i_t$ refers to agent *i*’s immediate reward at time step *t*.

Agents select actions based on the current state-action values $Q^i_t(s, a)$, balancing exploitation (i.e., selecting the action of maximum quality) and exploration (i.e., selecting lower quality actions in order to learn more about the environment). I here focus on the widely used Boltzmann policy. The probability of choosing action *a* in state *s* is

2 $$\begin{aligned} X^i_t(s, a) = \frac{e^{\beta Q^i_t(s, a)}}{\sum _b e^{\beta Q^i_t(s, b)} }, \end{aligned}$$

where the *intensity of choice* parameter $\beta$ controls the exploration–exploitation trade-off. Throughout this paper, I am interested in the learning process with fixed parameters $\alpha$, $\beta$ and $\gamma$ throughout learning and evaluating a policy.

## Deterministic learning dynamics

In this section, I first present a dense recap of the derivation of the deterministic discrete-time dynamic equations of temporal-difference reinforcement learning. A more elaborate version can be found in Ref. [7]. Then, I show how these equations relate to the higher-level properties of game-equilibira and free energy minimization. Note that temporal-difference learning generalizes simpler forms of stateless reinforcement learning used in many previous studies. In a stateless environment with discount factor $\gamma =0$, temporal-difference learning reduces to this simpler form of reinforcement learning. Thus, the presented results conceptually hold for these learning dynamics as well.

### Derivation

In essence, deterministic learning dynamics consider policy averages instead of individual realizations of rewards and state-action values. For multi-state multi-agent learning we need to find the average temporal-difference error ${{\bar{\delta }}}^i$ to be inserted in the update for the joint policy,

3 $$\begin{aligned} X^i_{t+1}(s, a) = \frac{X^i_t(s,a) \cdot \exp [\alpha \beta {{\bar{\delta }}}^i_t(s, a)]}{\sum _b X^i_t(s,b) \cdot \exp [\alpha \beta {{\bar{\delta }}}^i_t(s, b)] }, \end{aligned}$$

which can be derived combining Eqs. 1 and 2. Computing ${{\bar{\delta }}}^i$ involves averaging over policies and environmental transitions for all three terms of the temporal-difference error.

First, the average immediate reward becomes

$$\begin{aligned} {\bar{R}}^{i}(s, a) := \sum _{s'} \sum _{{\underline{a}}^{-i}} \underline{X}^{-i}(s, {\underline{a}}^{-i}) T(s, {\underline{a}}, s') R^i(s, {\underline{a}}, s') \end{aligned}$$

where $\sum _{s'} \sum _{{\underline{a}}^{-i}}$ ${\underline{X}}^{-i}(s, {\underline{a}}^{-i})$ $T(s, {\underline{a}}, s')$ is a short notation for

$$\begin{aligned}&\sum _{s'} \sum _{a^1} \cdots \sum _{a^{i-1}} \sum _{a^{i+1}} \cdots \sum _{a^N} X^1(s, a^1) \\&\quad \cdots X^{i-1}(s, a^{i-1}) X^{i+1}(s, a^{i+1}) \\&\quad \cdots X^{N}(s, a^{N}) T(s, {\underline{a}}, s'). \end{aligned}$$

Regarding notation, the bar over the symbol indicates that a policy average has been taken. When fewer state and action variables appear in the brackets, the remaining variables indicate which other variables have been used to average over.

Second, the quality of the next state, $\max _b Q^i_t(s_{t+1}, b)$, yields

$$\begin{aligned}&{}^\text {max}\!{\bar{Q}}^i(s, a) := \\&\quad \sum _{s'} \sum _{{\underline{a}}^{-i}} {\underline{X}}^{-i} (s, {\underline{a}}^{-i}) T(s, {\underline{a}}, s') \max _b {{\bar{Q}}}^i(s',b). \end{aligned}$$

Here, I replace the quality estimates $Q^i_t(s, a)$, which evolve in time *t* (Eq. 1), with the true state-action values ${{\bar{Q}}}^i(s, a)$, which is the expected return from executing action *a* in state *s* and then following along the joint policy ${\underline{X}}$. I compute ${{\bar{Q}}}^i(s, a) = (1-\gamma ) {{\bar{R}}}^i(s, a) + \gamma \sum _{s'}{{\bar{T}}}(s,a,s') {{\bar{V}}}^i(s')$, where ${{\bar{V}}}^i$ are the true state values. They are computed via matrix inversion according to ${{\bar{V}}}^i({\underline{s}}) = (1-\gamma ) [ \underline{\mathbb {1}}_Z - \gamma {{\bar{T}}}({\underline{s}}, {\underline{s}}) ]^{-1} {{\bar{R}}}({\underline{s}})$. Here, underlined state variables indicate that the corresponding object is a vector or matrix. $\bar{T}({\underline{s}}, {\underline{s}})$ indicates the policy-averaged transition matrix and the entry $\bar{T}(s, s')$ indicates the transition probability from state *s* to state $s'$. ${\bar{R}}^i(s)$ denotes the policy-average state rewards. $\underline{\mathbb {1}}_Z$ is a *Z*-by-*Z* identity matrix. Note, the quality ${}^\text {max}\!{\bar{Q}}^i(s, a)$ depends on *s* and *a* although it is the policy averaged maximum state-action value of the next state.

Third, the quality of the current state, $Q^i_t(s_t,a_t)$ becomes $\beta ^{-1} \ln X^i(s,a)$ in the average temporal-difference error and serves as regularization term. This can be derived by inverting Eq. 2 and realizing that the dynamics induced by Eq. 3 are invariant under additive transformations which are constant in actions.

All together, the average temporal-difference error for Q-learning reads

4 $$\begin{aligned} {{\bar{\delta }}}^i(s,a) = (1-\gamma ) {{\bar{R}}}^i(s, a) + \gamma {}^\text {max}\!{\bar{Q}}^i(s, a) - \frac{\ln X^i(s,a)}{\beta }. \end{aligned}$$

### Properties

Let us divide the average temporal-difference error into two parts, the non-regularized part plus the regularizer, ${{\bar{\delta }}}^i(s,a) = {{\bar{q}}}^i(s,a) - \ln X^i(s,a)/\beta$ with

$$\begin{aligned} {{\bar{q}}}^i(s,a) = (1-\gamma ) {{\bar{R}}}^i(s, a) + \gamma {}^{\text {max}}\!{{\bar{Q}}}^i(s, a). \end{aligned}$$

The deterministic map comprised of Eq. 3 and the non-regularized temporal-difference error,

$$\begin{aligned} X^i_{t+1}(s, a) = \frac{X^i_t(s,a) \cdot \exp {[\alpha \beta \bar{q}^i(s, a)]}}{\sum _b X^i_t(s,b) \exp {[\alpha \beta {{\bar{q}}}^i(s, b)]}} \end{aligned}$$

is equivalent to the alternative replicator dynamics in discrete time [48, 49] with $\alpha \beta {{\bar{q}}}^i(s,a)$ being the fitness of agent *i*’s action *a* in state *s*. This equivalence allows us to conjecture the folk theorem of evolutionary game theory [21, 49] for policy-average temporal-difference learning in stochastic games (a detailed proof is not the focus of this work and left for future work):

- A stable rest point is a Markov Perfect equilibrium.
- A convergent trajectory in the interior of the policy space evolves to a Markov Perfect equilibrium.
- A strict Markov Perfect equilibrium is locally asymptotically stable.

Roughly speaking, Markov Perfect equilibria can be seen as the Nash equilibria of the stochastic game when agents employ stationary Markov policies [26], i.e., as in this case, choose an action with a probability based only on the current state. This means no agent can gain more value by unilaterally changing its policy at a Markov Perfect equilibrium. Nontrivially, they have been shown to exist in discounted stochastic games with a finite number of agents [29].

Considering the dynamics with full average temporal-difference error including the regularization term,

5 $$\begin{aligned} X^i_{t+1}(s, a) = \frac{X^i_t(s,a) \cdot \exp {[\alpha \beta {{\bar{\delta }}}^i(s, a)]}}{\sum _b X^i_t(s,b) \exp {[\alpha \beta {{\bar{\delta }}}^i(s, b)]}}, \end{aligned}$$

the steady state policy, if it exists, can be obtained by setting ${{\bar{\delta }}}^i(s,a) = 0$ which yields after normalizing,

6 $$\begin{aligned} X^i_*(s,a) = \frac{e^{\beta {{\bar{q}}}_*^i(s,a)}}{\sum _b e^{\beta \bar{q}_*^i(s,b)}}. \end{aligned}$$

Despite their similarity, this set of equations is qualitatively different from Eq. 2; Eq. 6 gives a condition for the policies $X^i_*(s,a)$ and values ${{\bar{q}}}_*^i(s,a)$ at equilibrium.

One can show that the joint policy described in Eq. 6 follows from the principle of maximum entropy [53] under the constraint that agents play a policy the yields a constant expected value [114, 115]. The negative logarithm, $-\ln X^i(s, a)$, is called the information, surprise or uncertainty [91] of agent *i* taking action *a* in state *s*. The entropy $-\sum _b X^i(s,b)\ln X^i(s, b)$ is the average surprise of agent *i*’s policy in state *s*. The principle of maximizing the average surprise or uncertainty in a policy is reasonable whenever agents are not capable of computing the optimal policy straight away. This may because either agents are uncertain about their environment or they lack the cognitive resources. Within the context of active learning [89], this is also known as uncertainty sampling.

From the maximum entropy principle follows the principle of minimum free energy

$$\begin{aligned} F[{\underline{X}}^i(s)] = - \sum _b X^i(s, b) {{\bar{q}}}^i(s, b) + \frac{1}{\beta } \sum _b X^i(s,b) \ln X^i(s,b), \end{aligned}$$

which can be expressed as the action-mean policy-average temporal-difference error, $-F[{\underline{X}}^i(s)] = \sum _b X^i(s, b) {{\bar{\delta }}}^i(s,b)$. Minimizing the first term is equivalent to maximizing the expected average quality, minimizing the second term means maximizing the entropy of agent *i*’s choice of actions in state *s*. The Lagrange parameter $\beta$ balances these two contributions.

It is important to note that the minimum free energy principle does only hold for temporal-difference reinforcement learning when we differentiate the free energy with respect to the current action in the current state. In other words, we ask only for the effect of what the agent can do at this moment. We are not concerned with the effects of this action when the environment returns to the current state. This notion, that we are only interested in the effect of the current action *a* in state *s* resembles precisely the common definition of the state-action values ${{\bar{Q}}}^i(s, a)$ [98].

While the dynamics (Eq. 5) converge to a policy with minimum free energy (Eq. 6) one can also show that the steps the dynamics take minimize free energy differences,

7 $$\begin{aligned} \Delta F[{\underline{X}}_{t+1}^i(s)]&= F[{\underline{X}}_{t+1}^i(s)] - F[{\underline{X}}_{t}^i(s)] \nonumber \\&= - \sum _b X_{t+1}^i(s, b) \Delta {{\bar{q}}}^i(s, b) \nonumber \\&\quad \ + \frac{1}{\beta } \sum _b X_{t+1}^i(s,b) \frac{\ln X_{t+1}^i(s,b)}{\ln X_{t}^i(s,b)}, \end{aligned}$$

where $\Delta {{\bar{q}}}^i(s, a) := {{\bar{q}}}^i_{t+1}(s, a) - \bar{q}^i_t(s,a)$. Here I used the relationship $X^i_t(s, a) = e^{\beta {{\bar{q}}}_t^i(s, a)} / \sum _b e^{\beta {{\bar{q}}}_t^i(s,b)}$ analogous to Eq. 2. From this equality one can show that ${{\bar{q}}}^i_t(s, a) = \beta ^{-1}\ln X^i_t(s, a) + \beta ^{-1}\ln Z_t^i(s)$ with $Z_t^i(s) := \sum _b e^{\beta {{\bar{q}}}_t^i(s,b)}$ and $-\beta ^{-1} \ln Z_t^i(s) = F[{\underline{X}}^i_t(s)]$. Assuming the value differences between two steps $\Delta {{\bar{q}}}^i(s, a) = \alpha {{\bar{\delta }}}^i_t(s, a)$ such that ${{\bar{q}}}^i_{t+1}(s, a) = {{\bar{q}}}^i_t(s,a) + \alpha {{\bar{\delta }}}^i_t(s, a)$ (c.f. Eq. 1) it is exactly Eq. 5 that minimizes Eq. 7 [80].

## Algorithmic foundations

In order to connect the presented deterministic learning dynamics to an algorithmic level of analysis of lower abstraction, I present a temporal-difference reinforcement learning algorithm whose learning trajectories approach the ones of the deterministic dynamics. I will build up on the idea of batch learning that has been used in previous work on stateless reinforcement learning dynamics and extend it to multi-state temporal-difference learning. Originally, the batch reinforcement learning problem is defined to learn the best policy from a fixed set of a priori-known transition samples [60]. Research activity on batch reinforcement learning has grown substantially in recent years, primarily due to the central merits of the batch approach: (i) its efficient use of collected data and (ii) the stability of the learning process when used with function approximation. My focus though is not to present an efficient algorithm that solves the batch learning problem. Instead, I aim for an improved understanding regarding the algorithmic foundations underlying the policy-average temporal-difference learning equations.

I denote the proposed algorithm *sample-batch temporal-difference Q-learning*. The learning process is divided into two phases, an interaction phase and an adaptation phase. During the interaction phase, the agent keeps its policy fixed while interacting with its environment for *K* time steps, collecting state, action and reward information. During the adaptation phase, the agent uses the collected information for an update of its policy. Key is the use of two state-action value tables, one for acting (${}^{act}\!Q$), the other for improved value estimation (${}^{val}\!Q$). While ${}^{act}\!Q$ is kept constant during the interaction phase, ${}^{val}\!Q$ is iteratively updated.

During the interaction phase the information is collected as follows. The agent counts the number of visited state-action-next state triples,

$$\begin{aligned} t^i(s_t, a_t, s_{t+1}) \mathrel {+}= 1 \end{aligned}$$

and sums up immediate rewards for each state-action pair,

$$\begin{aligned} r^i(s_t, a_t) \mathrel {+}= r^i_t, \end{aligned}$$

with $r^i_t$ being the reward agent *i* received at time step *t*. Both, $t(s,a,s')$ and *r*(*s*, *a*) were initialized to zero for all $s,a,s'$. Further, each agent updates its value estimate of the current state-action pair as

8 $$\begin{aligned}&{}^{val}\!Q^i_{t+1}(s_t,a_t) \mathrel {+}\nonumber \\&\quad = \alpha \bigg [ (1-\gamma )r_t \ \nonumber \\&\qquad +\gamma \sum _b X^i_t(s_{t+1}, b) {}^{val}\!Q^i_{t+1}(s_{t+1}, b) \nonumber \\&\qquad - {}^{val}\!Q^i_{t}(s_t,a_t) \bigg ]. \end{aligned}$$

Note, I here do not use the off-policy $\max$ term in the value update. The aim of updating the ${}^{val}\!Q^i$ values is to estimate the true state-action values of policy $X^i$. The use of $\sum _b X^i_t(s_{t+1}, b) {}^{val}\!Q^i_{t+1}(s_{t+1}, b)$ is known as expected SARSA, which was shown to have significant advantages over other update schemes [54, 106]. At the beginning of each batch ${}^{val}\!Q^i_{t+1}(s, a)$ are set to ${}^{act}\!Q^i_{t+1}(s, a)$, which are initialized to $\ln (X_0^i(s, a))/\beta - \langle \ln (X_0^i(s, b))/\beta \rangle _b$. These interaction steps repeat until the current time step *t* is a multiple of the batch size *K*. Then the learner enters the adaptation phase.

During the adaptation phase, the agent uses the sample averages of the immediate rewards and next-state-value estimates to summarize the collected information in order to update the ${}^{act}\!Q$ values. For all states, *s*, and actions, *a*, the sample-average reward is computed as

$$\begin{aligned} {{\tilde{R}}}^i(s, a) = r^i(s,a) / d(s,a), \end{aligned}$$

with the divisor $d(s,a) = \min (1, \sum _{s'} t^i(s,a,s'))$. The divisor is used to avoid a division by zero, taking into account the possibility that a state-action pair might not have been visited. The sample-average transition model amounts to

$$\begin{aligned} {{\tilde{T}}}^i(s, a, s') = t^i(s,a,s') / d(s,a). \end{aligned}$$

The sample-average of the maximum next-value then yields

$$\begin{aligned} {}^{\text {max}}\!{\tilde{Q}}^i(s, a) = \sum _{s'} {{\tilde{T}}}^i(s, a, s') \max _b {}^{val}\!Q^i(s', b). \end{aligned}$$

This is the specific $\max Q$ update typically used in Q-learning. From these terms we can write the sample-average temporal-difference error as

$$\begin{aligned} {{\tilde{\delta }}}_t^i(s, a) = (1-\gamma ) {{\tilde{R}}}^i(s, a) + \gamma {}^\text {max}\!{\tilde{Q}}^i(s, a) - {}^{act}\!Q_t^i(s, a) \end{aligned}$$

Finally, the ${}^{act}\!Q$ values are updated according to

$$\begin{aligned} {}^{act}\!Q^i_{t+1}(s, a) = {}^{act}\!Q^i_t(s, a) + \alpha \cdot {{\tilde{\delta }}}_t^i(s, a), \end{aligned}$$

Last, the new policy is set to

$$\begin{aligned} X^i_{t+1}(s, a) = \frac{\exp [\beta \ {}^{act}\!Q^i_{t+1}(s, a)]}{ \sum _b \exp [\beta \ {}^{act}\!Q^i_{t+1}(s, b)]}, \end{aligned}$$

the values ${}^{val}\!Q_{t+1}^i(s, a) = {}^{act}\!Q_{t+1}^i(s, a)$, and $r^i(s,a)=0$, and $t^i(s,a,s') =0$, for all $s, a, s'$. Note that this sample-batch algorithm reduces to standard Q-learning for a batch size $K=1$.

## Results

In this section, I compare the deterministic learning equations (Sect. 3) with the sample-batch algorithm (Sect. 4) to show that their learning trajectories match under large batch sizes and thus, the sample-batch algorithm can be seen as an algorithmic foundation of the deterministic learning dynamics. I use two classes of environments as testbeds, a risk–reward dilemma and a zero-sum competition. For each comparison, I let both learning processes, deterministic equations and stochastic algorithm, update their policies 100 times. As a consequence, an increased batch size *K* results into an increased number of time steps as the sample-batch algorithm interacts with the environment.

### Risk-reward dilemma

The first environment I use as a testbed is a one-agent stochastic game, i.e., a Markov decision process. It is a simple, two-state, two-action environment and models the inter-temporal dilemma between a risky choice with possibly high immediate reward and a safe choice with a guaranteed, but low immediate reward [8]. The action set reads ${\mathcal {A}} = \{\mathsf {safe}, \mathsf {risky}\}$, the environment can either be in a prosperous or a degraded state, ${\mathcal {S}} =\{\mathsf {prosp.}, \mathsf {deg.}\}$. The transition function reads

$$\begin{aligned} T(\mathsf {prosp.}, a, \mathsf {deg.})= & {} \left\{ \begin{array}{ll} 0 &{} a=\mathsf {safe}\\ 0.2 &{} a=\mathsf {risky} \end{array}\right. , \\ T(\mathsf {deg.}, a, \mathsf {prosp.})= & {} \left\{ \begin{array}{ll} 0.1 &{} a=\mathsf {safe}\\ 0 &{} a=\mathsf {risky} \end{array}\right. . \end{aligned}$$

The reward function is given by

$$\begin{aligned} R(s, a, s') = \left\{ \begin{array}{ll} 1 &{} s=s'=\mathsf {prosp.} \ \text {and} \ a=\mathsf {risky} \\ 0.5 &{} s=s'=\mathsf {prosp.} \ \text {and} \ a=\mathsf {safe} \\ 0 &{} \text {elsewhere} \end{array}\right. . \end{aligned}$$

By applying the safe action in the prosperous state, the agent is guaranteed to remain in the prosperous state and obtains a reward of 0.5. If it applies the risky action and remains in the prosperous state, it obtains a reward of 1. If, however, the environment collapses under the risky action, the agent obtains 0 reward until the environment is recovered again. Recovery is possible only under the safe action after waiting for some iterations in the degraded state. In the prosperous state, it depends on the agent’s discount factor whether the risky or the safe action is more valuable to the agent.

**Fig. 2:**
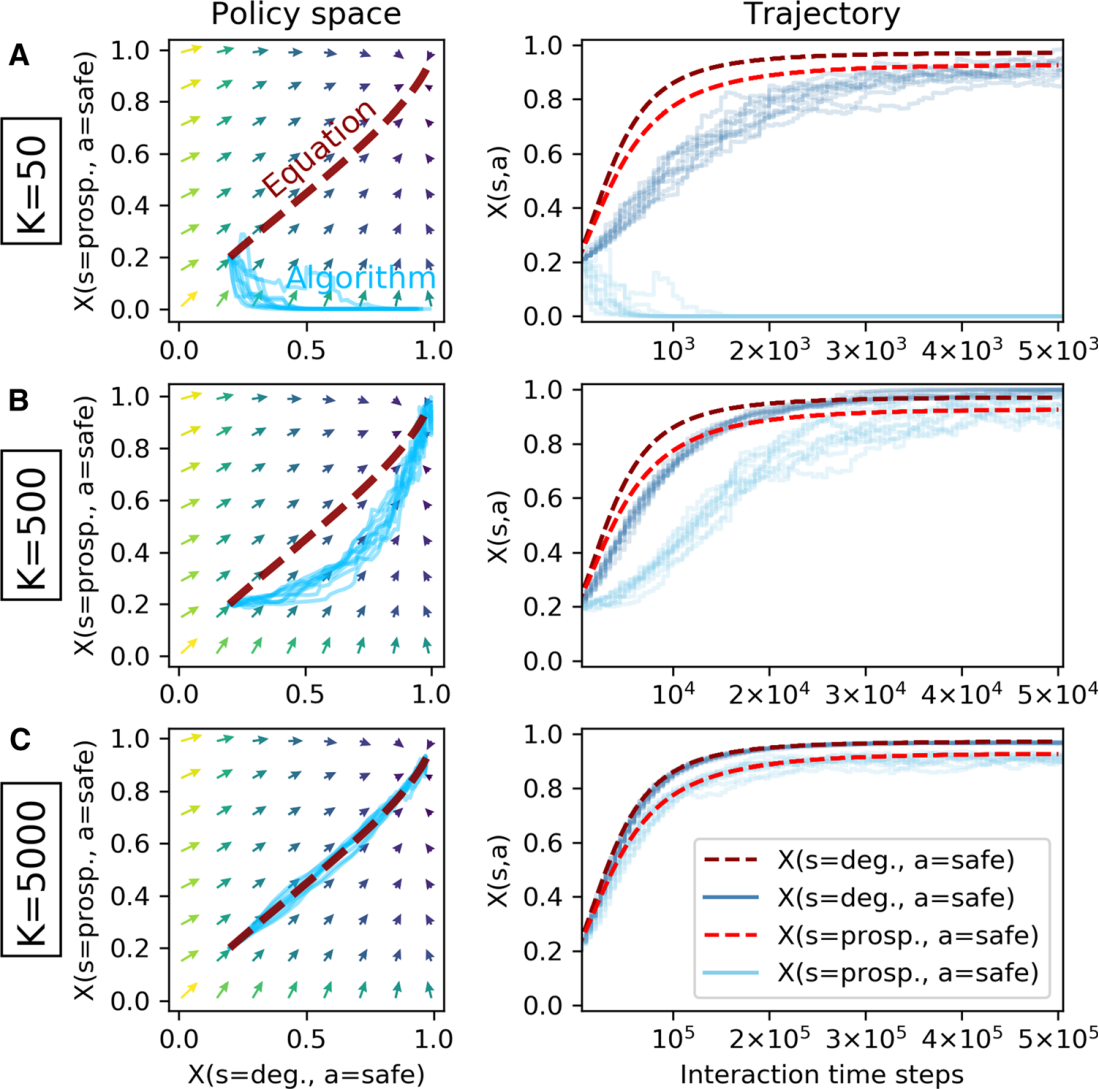
Single-agent risk–reward dilemma. Comparison between deterministic dynamics (dark red dashed line) with 10 runs of the sample-batch learning algorithm (light blue straight lines) for varying batch sizes *K* (**a**: $K=50$, **b**: $K=500$, **c**: $K=5000$). Agent parameters are $\alpha =0.05$, $\beta =150$, $\gamma =0.9$. The match between the sample-batch learning algorithm and the deterministic dynamics becomes increasingly closer as the batch size *K* increases

Figure 2 compares the deterministic dynamics with the sample-batch algorithm. For an agent with a discount factor of $\gamma =0.9$ it is optimal to select the safe action in both states. The challenge presented in Fig. 2 is learning to play safe in both states, starting from an initial policy where the safe action is chosen only with $20\%$ in both states. The arrows in the policy space indicate the average direction toward which the learner is driven by temporal-difference errors of the deterministic limit (Eq. 4). The deterministic dynamics (dark red dashed line) follow these temporal-difference arrows and learn to play almost completely safe. The small distance to the upper right corner in the policy space results from the finite exploitation parameter $\beta$.

Under increasing the sample-batch size *K*, the match between deterministic dynamics and sample-batch algorithm becomes increasingly closer. The sample-batch learning algorithm with batch size $K=50$ does not learn to play safe in the prosperous state (Fig. 2a). For a batch size of $K=500$, the agent learns to do so, yet, the trajectory through the policy space differs from the one of the deterministic limit (Fig. 2b). The agent learns to play safe in the degraded state faster than in the prosperous state. For a batch size of $K=5000$, algorithm and equations match almost perfectly (Fig. 2c).

Extending the single-agent risk–reward dilemma to a multi-agent environment, I let the agents face a classic social dilemma in the prosperous state [9]. Playing safe induces a costly contribution *c* to the public good from which all agents will receive a benefit *b*, regardless whether they contributed or not. However, each risky action will increase the probability by 0.2/*N* that the environment will collapse from the prosperous to the degraded state. In the degraded state, all agents receive a negative collapse impact $m <0$. Only the safe action will increase the recovery probability by 0.1/*N* from the degraded to the prosperous state. In previous work, we showed that the discount factor alone can transform this game from a tragedy of the commons, where the risky action dominates, into one of coordination, and even into a comedy of the commons in which the safe action dominates [9].

**Fig. 3:**
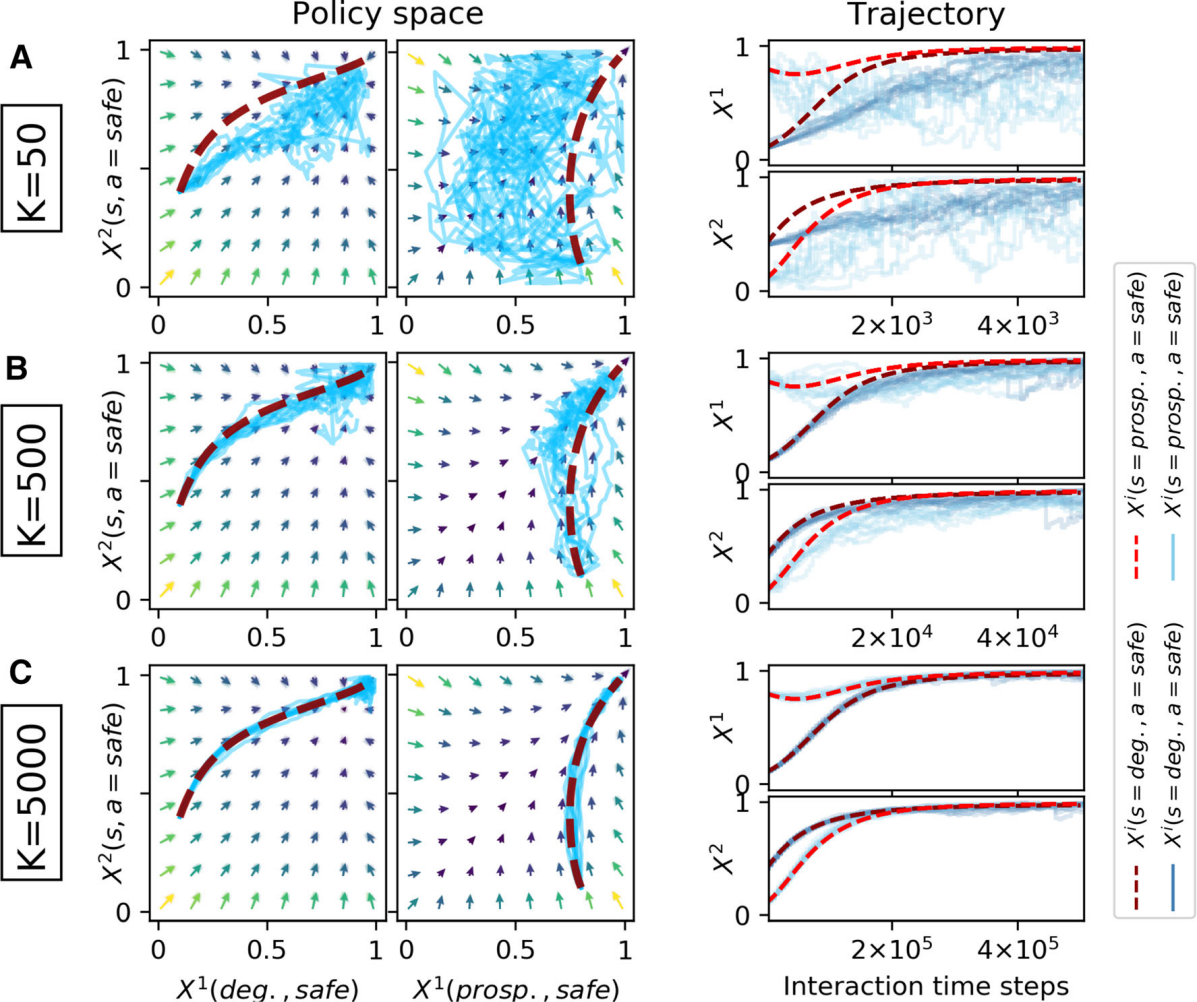
Two-agent risk–reward dilemma with contribution cost $c=5$, benefit $b=3$ and collapse impact $m=-5$. The deterministic dynamics (dark red dashed line) are compared against 10 runs of the sample-batch learning algorithm (light blue straight lines) for varying batch sizes *K* (A: $K=50$, B: $K=500$, C: $K=5000$). Agent parameters are $\alpha =0.04$, $\beta =25$, $\gamma =0.9$. The match between the sample-batch learning algorithm and the deterministic dynamics becomes increasingly closer as the batch size *K* increases

Figure 3 compares the deterministic dynamics with the sample-batch algorithm for an environment with 2 agents. Parameters are as such that the game is a comedy, where the game-equilibrium corresponds to all agents playing safe in both states. And indeed, the deterministic dynamics (dark red dashed line) reach this policy from the asymmetric initial policy, where agent 1 plays safe with 10% in the degraded and 80% in the prosperous state and agent 2 play safe with 40% in the degraded and 10% in the prosperous state.

Here too, as well, the match between deterministic dynamics and sample-batch algorithm becomes increasingly closer under increasing sample-batch size *K*. For $K=50$, the learning behavior of the algorithm in the prosperous state appears to be random around the center of the policy space (Fig. 3 a). For a batch size of $K=500$, algorithm and deterministic dynamics match already well (Fig. 3 b). The match is perfected for $K=5000$ (Fig. 3 c).

### Zero-sum competition

The other environment I use as a testbed is the two-agent ($N=2$), two-state (${\mathcal {S}} = \{1, 2\}$), two-action ($\mathcal A=\{\mathsf {left}, \mathsf {right}\}$) matching pennies game [40]. It roughly models the situation of penalty kicks between a kicker and a keeper. Both agents can choose between the left and the right side of the goal. The keeper agent scores one point if it catches the ball, otherwise the kicker agent receives one point. Agents change roles under state transitions, which depend only on agent 1’s actions. Precisely when agent 1 selects either left as keeper or right as kicker both agent will change roles. With symmetrical rewards but asymmetrical state transitions, the two-state Matching Pennies game presents the challenge of coordinating both agents on playing a mixed strategy with equiprobable actions.

**Fig. 4:**
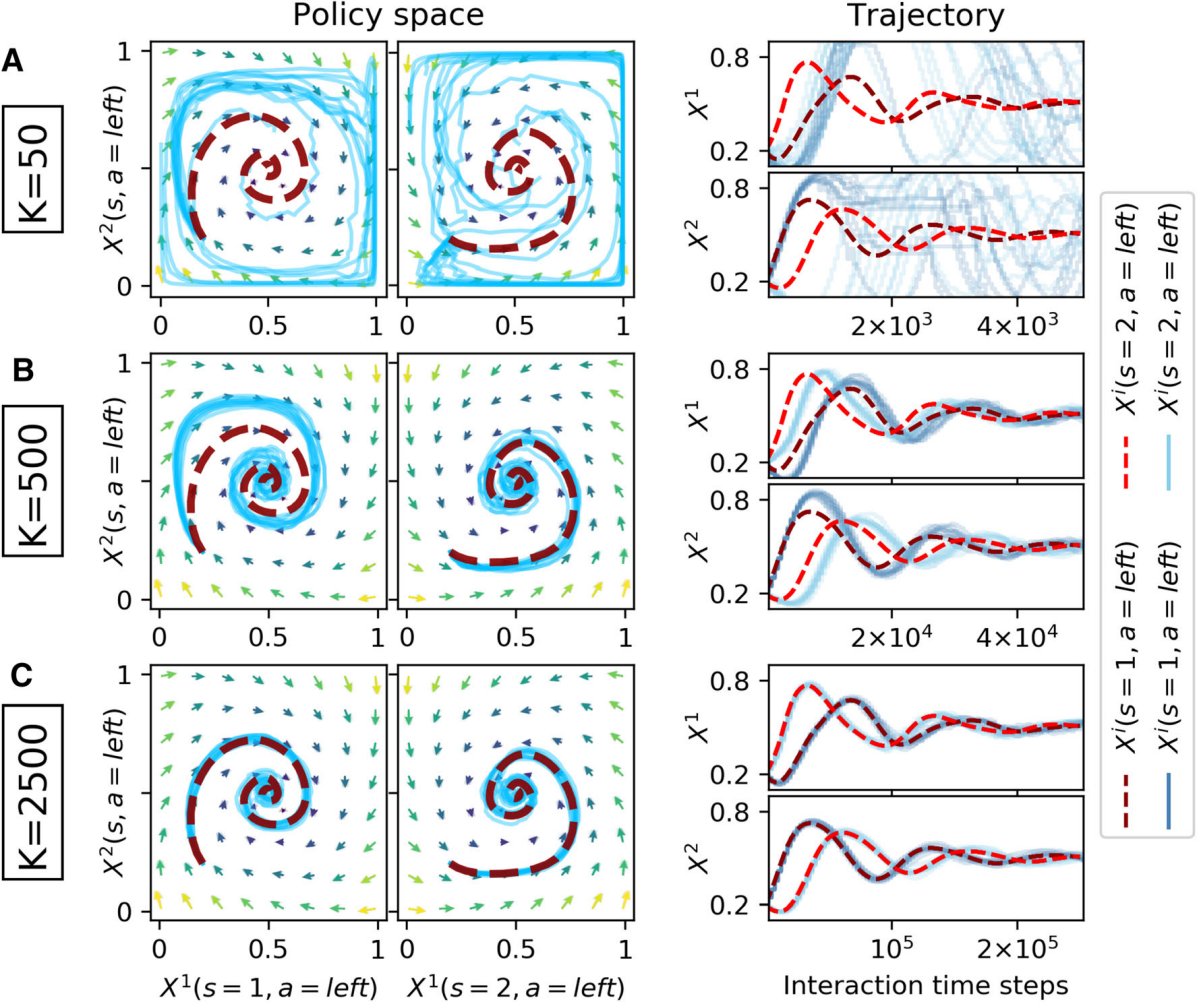
Zero-sum competition with the two-state Matching Pennies game. The deterministic dynamics (dark red-dashed line) are compared against 10 runs of the sample-batch learning algorithm (light blue straight lines) for varying batch sizes *K* (**a**: $K=50$, **b**: $K=500$, **c**: $K=2500$). Agent parameters are $\alpha =0.05, \beta =25, \gamma =0.75$. The match between the sample-batch learning algorithm and the deterministic dynamics becomes increasingly closer as the batch size *K* increases

Figure 4 shows that the match between deterministic dynamics and sample-batch algorithm becomes increasingly closer under increasing sample-batch size *K*. The deterministic dynamics (dark red-dashed line) manage to find this mixed Markov equilibrium in the center of the policy space from a spiraling learning trajectory starting with both agents selecting left with 20% in both states. The sample-batch algorithm with batch size $K=50$ does not capture the learning trajectory of the deterministic dynamics (Fig. 4a). For a batch size of $K=500$, algorithm and deterministic dynamics match already well (Fig. 4b). The match is perfected for $K=5000$ (Fig. 4c).

**Fig. 5:**
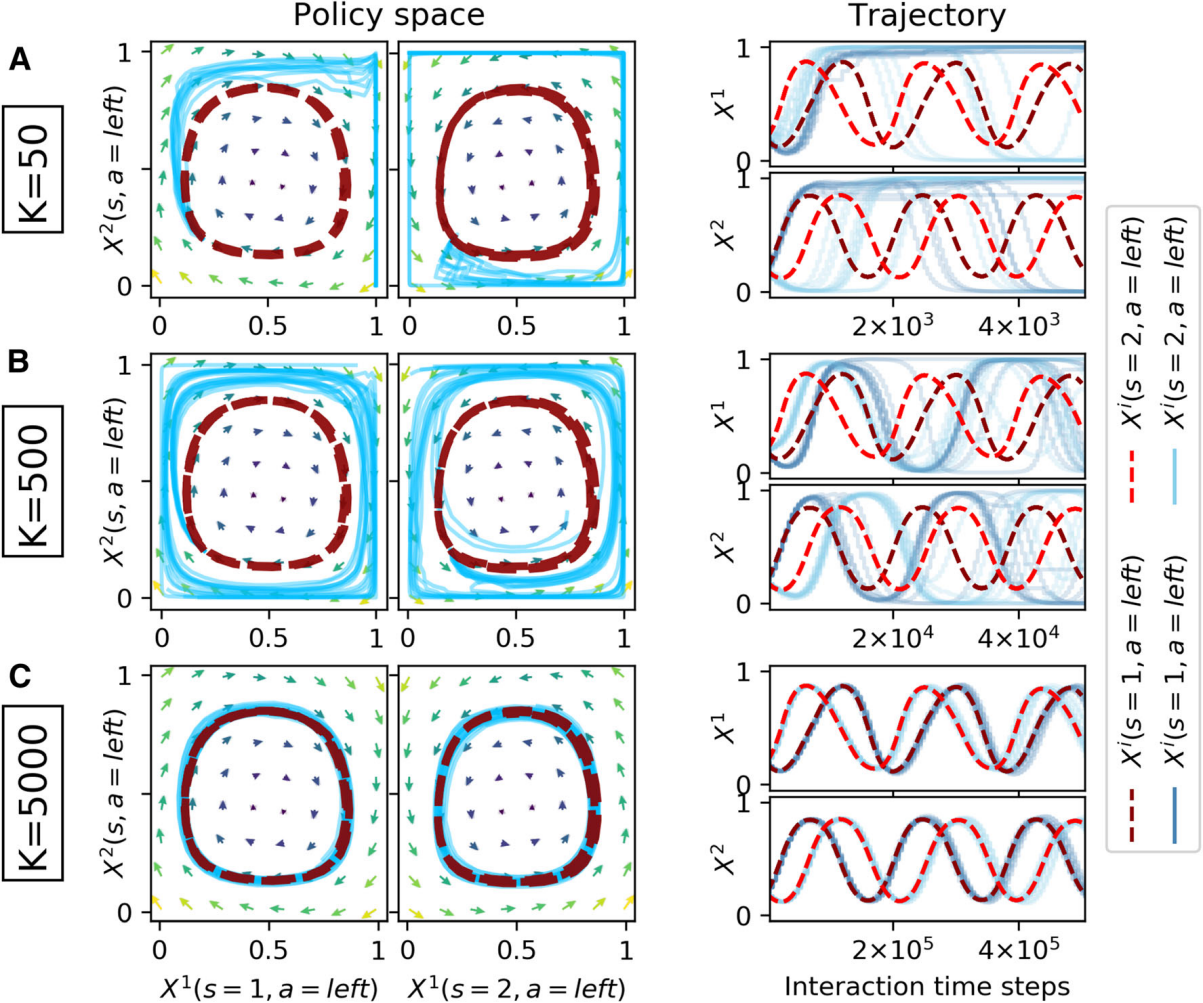
Zero-sum competition with the two-state Matching Pennies game. The deterministic dynamics (dark red dashed line) are compared against 10 runs of the sample-batch learning algorithm (light blue straight lines) for varying batch sizes *K* (**a**: $K=50$, **b**: $K=500$, **c**: $K=5000$). Agent parameters are $\alpha =0.02, \beta =45, \gamma =0.55$. Here, the deterministic dynamics learn on a periodic orbit in policy space. Nevertheless, the sample-batch learning algorithm matches the deterministic dynamics under a sufficiently large batch size *K*

Figure 5 shows that the sample-batch algorithm approaches the deterministic dynamics under increasing sample-batch size *K* even for parameter domains at which the deterministic agents no longer converge to a fixed point policy but instead learn on a periodic orbit of changing policies.

### Rate of convergence

**Fig. 6:**
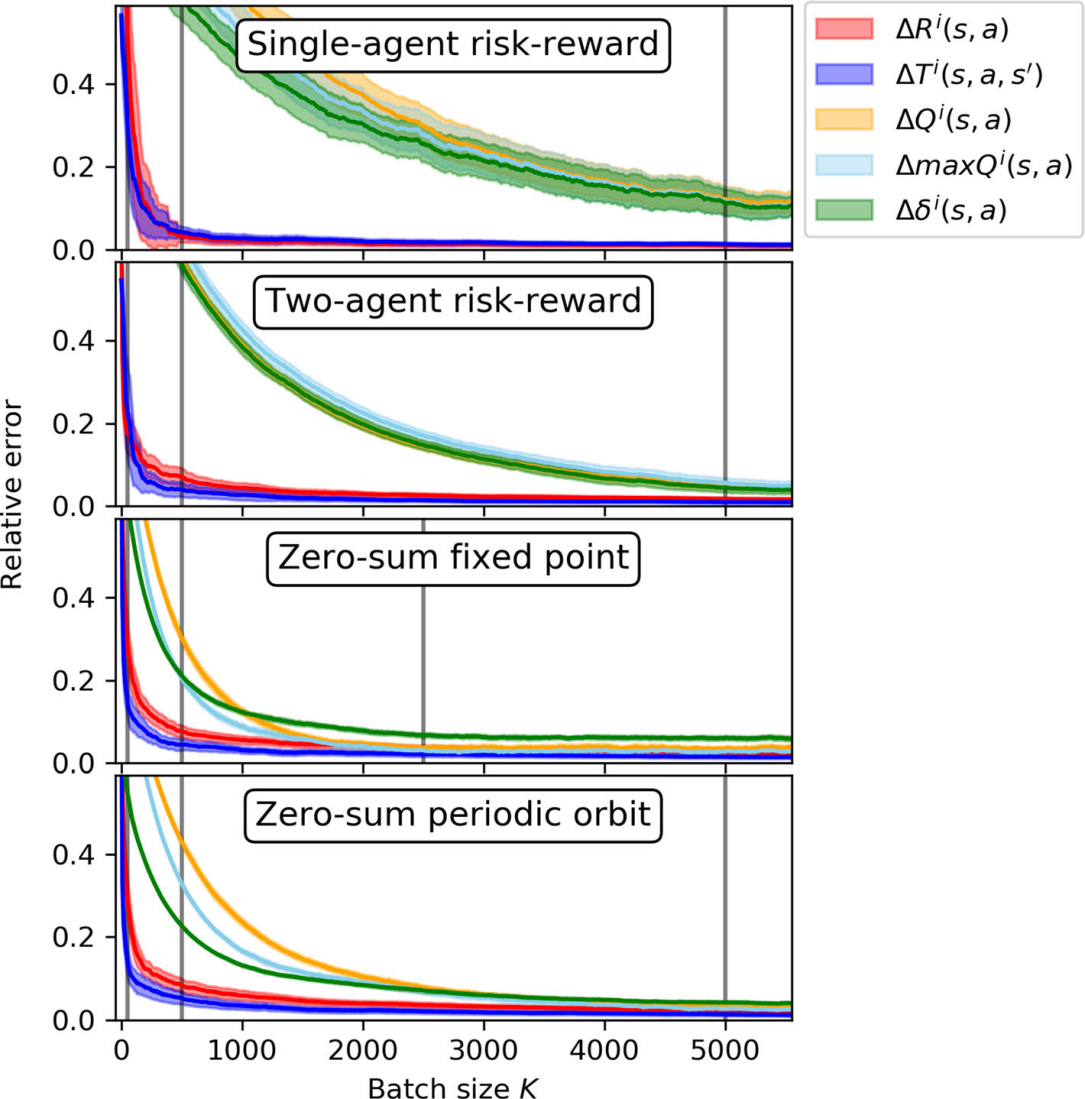
Relative error of temporal-difference error terms (reward $\Delta R^i(s, a)$ in red, transitions $\Delta T^i(s, a, s')$ in dark blue, values $\Delta Q^i(s, a)$ in orange, max values $\Delta {}^\text {max}Q^i(s,a)$ in light blue, temporal-difference error $\Delta \delta ^i(s,a)$ in green) versus the batch size *K* for the four environments presented (single-agent risk–reward environment in Fig. 2, two-agent risk–reward environment in Fig. 3, zero-sum competition with fixed point in Fig. 4, zero-sum competition with periodic orbits in Fig. 5). Shown are the averages of 100 sample runs in the center of on standard deviation. Each plot uses the initial policy from its corresponding Figs. 2-5. Vertical lines indicate the batch size shown in those figures

So far, I showed that the sample-batch algorithm discussed in Sect. 4 matches the deterministic dynamics derived in Sect. 3 under sufficiently large batch sizes. From Eq. 3, it is clear that the reason for this match is the temporal-difference error. Under large batch sizes the temporal-difference error of the algorithm must converge to the average temporal-difference error (Eq. 4). To get a better understanding of the rate of this convergence, I investigate the individual contributions of the temporal-difference error in more detail. Specifically, I consider the average rewards $\bar{R}^i(s, a)$, the environmental transitions ${{\bar{T}}}^i(s, a, s')$, the state-action values ${{\bar{Q}}}^i(s, a)$, the maximum state-action values ${}^\text {max}{{\bar{Q}}}^i(s,a)$, and finally the full temporal-difference errors ${{\bar{\delta }}}^i(s,a)$. For each of those terms, I compute the relative error between the algorithmic value and the deterministic value. For an average temporal-difference error term ${{\bar{Y}}} \in \{{{\bar{R}}}^i(s, a), {{\bar{T}}}^i(s, a, s'), \bar{Q}^i(s, a), {}^\text {max}{{\bar{Q}}}^i(s,a), {{\bar{\delta }}}^i(s,a)\}$ with the corresponding algorithmic value ${{\tilde{Y}}}(K)$ for batch size *K*, I define the relative error to be

9 $$\begin{aligned} \Delta Y(K) = \left\langle \left| \frac{{{\tilde{Y}}}(K) - {{\bar{Y}}}}{ \langle {{\bar{Y}}}\rangle _{s,a,s'}}\right| \right\rangle _{s,a,s'} \end{aligned}$$

where I use $\langle . \rangle _{s,a,s'}$ to take the sample average along all states *s*, actions *a* and next state $s'$, if they exist in the temporal-difference error term *Y*. Fig. 6 shows how the relative errors of the temporal-difference error terms depend on the batch size *K*. Since the algorithm is stochastic, I computed 100 sample runs and plot one standard deviation around the mean.

Across all environments, we observe two qualitatively different processes of convergences. Compared to the other terms, rewards ${{\tilde{R}}}^i(s,a)$ and transitions ${{\tilde{T}}}^i(s,a,s')$ converge much faster. Their convergence is a direct consequence of the central limit theorem and their convergence is thus proportional to the inverse square root of the batch size, $1/\sqrt{K}$. The other terms, the state-action values ${{\tilde{Q}}}^i(s, a)$, the maximum state-action values ${}^\text {max}{{\tilde{Q}}}^i(s,a)$ and the full temporal-difference errors ${{\tilde{\delta }}}^i(s,a)$, all depend on the value estimation (Eq. 8). This processes convergences slower than $1/\sqrt{K}$ in all environments. Yet, here differences between environment classes exist. The convergence of the value terms occurs faster in the zero-sum environments than in the risk–reward dilemmas. One explanation for this phenomenon is the environmental dynamics. In the zero-sum games, state changes occur more often than in the risk–reward dilemmas, allowing for a faster and smoother convergence.

Overall, Fig. 6 explains the results shown in Figs. 2, 3, 4,5. For batch sizes of $K=50$ and $K=500$, the differences between algorithmic and average temporal-difference errors are simply too large for a close match between algorithmic and determinsitic learning trajectories through policy space. For the environments used in this article, a batch size of $K=5000$ is able to let the learning trajectories match well. Interestingly, the quality of this match must depend also on the type of the attractor. For agent parameters that lead to a periodic orbit in the zero-sum environment, a batch size of $K=5000$ is required to let the learning trajectories match well. For agent parameters that let the learning convergence to the center of the policy space, a good match of the learning trajectories could already be achieved with $K=2500$. The reason is that the center of the policy space does not require the same amount of precision about where to go next. On a periodic orbit, small errors can propagate more easily and thus can lead to different learning trajectories in policy space.

## Discussion

In this article, I proposed to regard the replicator reinforcement learning dynamics perspective on multi-agent learning as a level of cognitive analysis. This dynamical systems level still has a high level of abstraction, connecting the computational level of the problem statement with lower algorithmic levels that aim to find a solution for the computational problem. I argue that it are the connections between those levels that can bring improved insights about the whole system of multiple learning agents (Fig. 1). I demonstrated this approach with the classic model-free independent temporal-difference Q-learning algorithm. It generalizes simpler forms of reinforcement learning used in previous studies and has importance also in neuroscience through the reward-prediction hypothesis [87]. I first discuss specifics, limitations and related work about the dynamic equations and the proposed sample-batch algorithm separately, before I highlight how the learning dynamics form a model of bounded rationality which can be used as a cognitive interpretation of individual learning in models of social dynamics. I finish with an outline for future work.

### Dynamics

With respect to the connection between the computational level and the dynamical systems level of learning, I have shown that the equilibria of these dynamics, if they exist, minimize a free energy functional resulting from a maximum entropy approach. Further, I showed that the discrete time dynamics minimize free energy differences at each learning step.

The concept of free energy minimization occurs in various other related domains. Minimizing free energy differences has been proposed as a thermodynamic theory of decision-making with information processing costs, which encompasses optimal, risk-sensitive and robust decision-making as special cases [79, 80]. Also, free energy minimization has served as a theory of boundedly rational decision-making [114, 115]. Eq. 6 resembles Quantal Response [74, 75] or logit equilibria [4] from behavioral game theory, which are able to provide a unified perspective on many of the systematic deviations from Nash equilibria in behavioral experiments. For example, the famous Ellsberg’s and Allais’ paradoxes can be explained [80]. Under a particular choice of rewards, which minimize the agents’ observational surprise, minimizing the free energy approximates Bayesian inference [67, 80]. Such a variational Bayes approach has also been proposed as a unified theory to understand brain function [30]. With respect to the domain of optimal control, closely related ideas are entropy regularization [77], reinforcement learning as probabilistic inference [62], maximum-entropy reinforcement learning [119], energy-based reinforcement learning [39, 84] and Kullback–Leibler divergence minimization [36, 57, 101].

It is interesting to observe the different roles the parameter $\beta$ receives from these different contexts. From decision-making under uncertainty, the intensity of choice $\beta$ regulates the exploration–exploitation trade-off. In the min free energy principle $\beta$ is a Lagrange multiplier. In boundedly rational decision-making $\beta$ regulates the degree of an agent’s rationality or likewise $\beta ^{-1}$ the cognitive cost of the agent. Indeed, $\beta ^{-1}$ must come in units of reward per units of information, i.e., how expensive it is for an agent to acquire information about the optimal policy. For decision-making under uncertainty, $\beta ^{-1}$ expresses the value of being unsure about what to do. If the agent knows nothing about the environment being unsure about what to do is more valuable compared to the situation when the agent already learned something about the environment. This suggests the use of an adaptive $\beta$ [102].

### Algorithm

In order to connect the dynamical systems level to an algorithmic level of lower abstraction, I proposed a simple batch learning algorithm for temporal-difference Q-learning. I showed that under increasing batch size the learning trajectories of this sample-batch algorithm match the ones of the deterministic equations. Two characteristics of the algorithm are essential for this property. First, the policy is updated using a batch of experiences, i.e., state-action transitions. Second, the state-action values are separated into two data structures, one for the value estimation inside the batch, the other for acting outside the batch.

This algorithms has various similarities with well-known algorithms from the literature. For one, its structure is similar to the influential *DQN*-paradigm [76], which also combines the replay of a batch of experiences with separated state-action value estimation. This suggests that some of DQN’s success might be due to the fact that it approximates a free-energy minimizing learning dynamic whose stable rest points correspond to Nash equilibria under $\beta \rightarrow \infty$. Nevertheless, significant differences remain. Most importantly, DQN is used with non-linear function approximation, whereas my algorithm is a tabular method. Being in the tabular setting is certainly a limitation of my work. Both, algorithm and dynamic equations do not scale to more complex environments.

With respect to use of separate state-action value estimation, a related approach is *double-Q* learning [38]. It uses two Q-tables to avoid an overestimation of the next state value caused by the *max* operator used in Q-learning. One Q-table is used for an update of the other and vice versa. In contrast, my algorithm’s Q-tables keep their roles as actor and value-estimator fixed. Overestimation is avoided by a large batch sizes which lead to unbiased policy-average estimators.

With respect to use of a batch for memory replay, my sample-batch algorithm has similarities with a stochastic *Dyna* agent [97] that uses experienced reward and state samples to simultaneously update a value function and learn a model. Indeed, the distinction between *model-free* and *model-based* algorithms is not always clear and memory replay blurs the line between model-based and model-free methods [107]. The data structures of the proposed algorithm, which store rewards and transitions, are the model the agent learns while interacting with the environment. The state-action values for improved value estimation inside the batch (${}^{\text {val}}\!Q$) are simultaneously updated. The use of the current policy of my algorithm resembles what is known as trajectory sampling [11]. In contrast to Dyna, the model is learned for each policy from scratch and also no reward-state transitions are sampled from the model. This makes the proposed algorithm highly data-inefficient. Yet, my goal was not to create an efficient algorithm, but to improve on the understanding of the deterministic temporal-difference learning dynamics.

All related algorithm discussed so far fall under the category of *value-based* or *critic-only* methods, that aim to obtain a good estimate of the state-action value function first and then derive a policy from it. However, the use of the soft-max policy in combination with the two Q-tables has also similarities with the family of *actor-critic algorithms* [59] and *policy gradient methods* [99]. One Q-table is used for acting (${}^{\text {act}}\!Q$), the other for improved values estimation and criticizing the actor (${}^{\text {val}}\!Q$). Actor-critic algorithms have also been referred to as two-time-scale algorithms [59] in which the actor is updated on a slower time scale than the critic. This description matches my sample-batch algorithm perfectly. One advantage of actor-critic methods is their capability of learning a stochastic policy explicitly, which turns out to be useful in competitive and non-Markov cases [94]. And indeed, we have seen the convergence of both the sample-batch algorithm and the learning equations in the zero-sum competition (Fig. 4).

Taken together, the classification of the replicator reinforcement learning dynamics need revision. Although originally derived from model-free temporal-difference reinforcement learning algorithms [7], they should be classified as model-based learners. In the limit of an infinite memory-batch, they obtained a perfect model of their current environment. However, the environmental transitions each agent learns are influenced by the policies of the other agents. And since this influence of the other agents’ policies is taken into account perfectly by the deterministic equations, i.e., they condition their perfect model of the environment on the current policies of the other agents without error, they should also be classified as joint-action space learners. This is interesting because originally, the deterministic learning dynamics were derived from a set of independent temporal-difference learning algorithms.

### Bounded rationality

We can use those deterministic dynamics for a boundedly rational account of game theory in two ways. First, added cognitive costs and second, agents no longer assume that other agents behave rationally.

First, the idea to model a cognitive cost of computation through a Boltzman softmax function is nothing new [4, 115]. Yet, I have shown that the Boltzman softmax function can originate from the need to regulate the exploration–exploitation trade-off in a decision-making situation under uncertainty. To unify both perspectives, bounded rationality and decision-making under uncertainty, the corresponding parameter $\beta$ might be called the *ecological rationality* parameter. Generally, the study of ecological rationality uncovers the conditions of how computationally bounded minds use simple heuristics and produce accurate decisions by exploiting the structures of information in uncertain environments [100]. Thus, on a high level of abstraction, the exploration–exploitation trade-off justifies bounded rational decision-making and explains why it can be beneficial in decision-making contexts under uncertainty. The best choice of the ecological rationality $\beta$ depends on the environment. In this sense, multi-agent learning dynamics can serve also as a high-level theory of ecological rationality.

Second, the classic solution to a game in the form of a Nash equilibrium requires the assumptions that all agents behave fully rational and have complete information about rewards and environmental transitions. Thus, all agents assume all other agents to behave rationally as well. In contrast, the deterministic learning equations use only the other agents’ current policy to update their own policy. As a result, learning behavior emerges in policy space, instead of game-equilibrium points. Such learning dynamics help overcome the game-equilibrium selection problem, if more than one such equilibrium exists.

In combination, learning dynamics with full cognitive rationality, i.e. the extreme case of $\beta \rightarrow \infty$ (under $\alpha \beta = 1$), the deterministic dynamics approach the alternative replicator dynamics in discrete time. For the replicator dynamics, the folk theorem of evolutionary game theory connects dynamic equilibria to game-theoretic equilibria. Stable rest points of the dynamics correspond to Nash equilibria of the game. However, this only works because the deterministic dynamics learn as if they have a perfect model of the environment and the other agent’s current policy and thus, they are not required to explore the environment with some finite $\beta$. Additionally, with temporal-difference learning we are not restricted to normal-form games. Instead, the more general form of stochastic games allows us to investigate the interactions of multiple agents within a changing environment [9, 47].

**Table 1:** Differing agent components at different analysis levels of multi-agent learning

	Model of environment	Model of other agents	Cognitive costs	Dynamics
Game-equilibria	Perfect	Rational agents	None	None
Deterministic learning dynamics	Perfect	Current policy	Stylized	Deterministic
Sample-batch learning algorithm	None	None	Algorithmic	Stochastic

This account of boundedly rational game theory can also be embedded within the framework of cognitive levels of analysis (Table 1). The stochastic game corresponds to the upper level of the computational problem that is posed to the agents. Typically, it is assumed that agents have perfect information about their environment, assume that all other agents behave rationally as well, require no cognitive cost of computation and thus find some static equilibrium. The learning dynamics correspond to an algorithmic level with a high level of abstraction. Here, agents are assumed to have a perfect model of the environment and of the other agents current policy. Cognitive costs are included in the model and tunable by the parameter $\beta$. The outcome is a deterministic dynamic learning trajectory which is not necessarily converging to a rest point. The proposed learning algorithm corresponds to an algorithmic level of lower abstraction. Agents require no knowledge about the environment in advance. They learn a model of the environment and the other agents current policy through repeated interactions with the environment and each other under the same policy. The cognitive costs of computing this model are immense and dealing with the exploration–exploitation is required. As a result, the agents’ learning is stochastic.

### Future work and potential applications

The proposed algorithm is not resource-efficient at all. In fact, the computational costs are infinite to match the deterministic learning dynamics exactly. Future work is needed to advance the link between the deterministic learning dynamics and more data-efficient algorithms toward resource-rational analysis. A natural start to do so is to not erase all collected experience after a policy update and instead start from the current reward and transition model. The equations need to be extended to account for imperfect models by adding suitable noise terms to the temporal-difference error, building upon previous work on stateless learning dynamics [32]. Thereby, it is important to investigate the conditions influencing the rate of convergence of the temporal-difference error. The convergence quality surely must depend on the environmental dynamics. State that are visited rarely will correspond to increased noise.

*Potential applications.* The improved insights about the connections between different analysis levels may have potential use with respect to the following application domains.

*Hyper-parameter tuning.* How does the quality and dynamics depend on hyper-parameters of the algorithm and features of the environment? For example, the size of the memory buffer in experience replay needs careful tuning [118]. A noisy version of the replicator reinforcement learning dynamics may shed light on the principles for determining decent batch sizes that can be translated to learners in complex environments.

*Design of new algorithms.* Can we reduce the number of hyper-parameters by replacing them through sensible functional relationships? For example, an inexperienced agent should explore more than an experienced one. The level of experience of an agent should also depend on the discount factor. An agent with a small outlook on the future should exploit faster than an agent who values the future more. Future work may investigate and formalize those verbal principles and convert its findings into novel algorithms. Based on this work, these algorithms can be deployed and tested both on the idealized process level of replicator reinforcement learning dynamics as well as on the concrete process model of reinforcement learning algorithms. Additionally, extensions to the basic reinforcement learning update scheme, such as *leniency* [81] and *Win-or-Learn-Fast (WoLF)* [16], could be rapidly prototyped on the idealized process level and then converted to the concrete process algorithms.

*Analysis of strategic interactions.* How do hyper-parameter combinations affect the resulting collective incentive structure in relation to features of the environment? For example, in previous work, we found that a sufficiently large discount factor can change a tragedy of the commons into a comedy of the commons where the mutually beneficial cooperative action dominates, given a potential environmental threat is sufficiently severe [9]. We must assume that this found principle also conveys to more complex environments. An interesting area for future work is to study the influence of the batch size in relation to the other hyper-parameters on the emerging incentive structure.

*Modeling of social dynamics.* Related to the analysis of strategic interactions, replicator reinforcement learning dynamics are of potential use in models of social dynamics that apply evolutionary game theory to human social phenomena [2, 25, 46, 69, 73, 85, 108, 110]. Methodologically, the use of evolutionary game theory can be seen as a middle ground between the highly formal classic game theoretic equilibrium analyses and more open agent-based-modeling approaches. It has been justified either as as a theoretical tool to identify robust behavioral policies or as model of social imitation learning. This work provides a third interpretation for studying social dynamics. Reinforcement learning agents learn on their own in a boundedly rational fashion. This interpretation might be valuable especially when a social learning interpretation might not be justifiably from the modeling context.

## Conclusion

Evolutionary game theory is a fruitful framework to enhance the interpretability of multi-agent learning systems. In this work, I have enhanced the interpretability of the link between evolutionary game theory and reinforcement learning itself.

Conceptually, I have embedded replicator reinforcement learning dynamics into different levels of cognitive analysis. Doing so allowed me to investigate the connections between the dynamical systems level to the computational level and algorithmic levels of lower abstraction. I have found that temporal-difference replicator reinforcement learning dynamics follow a principle of minimum free energy and combines a boundedly rational account to game equilibria in stochastic games with decision-making under uncertainty. I have introduced a sample-batch algorithm and showed that it serves as a micro-foundation for the replicator reinforcement learning equation. The algorithm combines memory-batch learning with separated state-action value estimation. The learning trajectories of the algorithm matches the ones of the equation under a large memory-batch, which I have empirically shown across two classes of environments.

Taken together, the classification for replicator reinforcement learning dynamics must be revised. Although originally derived from independent, model-free temporal-difference reinforcement learning algorithms, replicator reinforcement learning dynamics should be classified as model-based, joint-action learners. They use a perfect model of the environment and the other agents’ current policies balancing the rewards from the environment with the exploration–exploitation costs of cognition.

## Data Availability

Not applicable

## Declarations

### Code availability

Python code to generate all reported results is available at Zenodo (10.5281/zenodo.4982727) and GitHub (https://github.com/wbarfuss/CognitiveLevels).
